# Environmental policy-making networks and the future of the Amazon

**DOI:** 10.1098/rstb.2007.0038

**Published:** 2008-02-11

**Authors:** Maria Carmen Lemos, J. Timmons Roberts

**Affiliations:** 1 James Martin 21st Century Fellows, Environmental Change Institute, Oxford University Centre for the EnvironmentOxford OX1 3QY, UK; 2 School of Natural Resources and Environment, University of MichiganDana Building, 440 Church Street, Ann Arbor, MI 48109-1041, USA; 3 Environmental Science and Policy Program, The College of William and MaryWilliamsburg, VA 23187, USA

**Keywords:** Amazon, compensated reduction, avoided deforestation, Planafloro, policy networks, Brazil

## Abstract

This article examines four periods of environmental policy-making in the Amazon region of Brazil. It specifically analyses the role of pro-environment and pro-development policy networks in affecting policy design and implementation. It argues that the efforts of environmentalist networks trying to advocate or block relative developmentalist policies in the Amazon depend on three critical factors—whether they are able to attract the support of elites (or at least block their developmentalist policy initiatives); the type and level of international support they have; and the organizational and financial resources that they are able to mobilize. In analysing the four periods, this article finds that while international influences and resources have been substantial in enabling environmentalist networks to flourish and influence the policy, their effectiveness has been nearly always outweighed by Brazilian developmentalist interests. The outcome in each phase has been a different form of *stalemate* on environmental protection, and the deforestation continued each time, albeit at slower rates. These findings suggest that the key for significantly lower rates of deforestation on the Amazon may be in the ability of pro-environment networks to neutralize opposition by creating an incentive structure that ‘compensates’ potential losers of policies that promote conservation.

## 1. Introduction

The rate at which Amazon forests are destroyed is the result of many factors, including economic trends and incentives, population migration and growth, urbanization, property rights and enforcement of environmental laws. Several of these are the direct result of a complex and pitched struggle over the formation and implementation of land- and resource-use policies of the region. This paper focuses on the efforts of policy networks trying to advocate or block relatively developmentalist or environmentalist policies in the Amazon in light of three critical factors—whether they are able to attract or neutralize domestic elites; the type and level of international support they have; and the organizational and financial resources they are able to mobilize. These factors largely determine whether these networks succeed or fail. For analytical purposes, we divide these networks into two camps, named as pro-environment and pro-development; keeping in mind that the depiction of the struggle over policy-making in the Amazon as a dichotomy between these two networks is oversimplification of a complex and heterogeneous, socioecological system in which conflicting interests and fluid allegiances form and reform depending on the issue galvanizing mobilization. Within the pro-environmental network, for example, actors may disagree about their support to indigenous and other ‘social’ causes or the different strategies to foster conservation cum development, but agree to the need for measures that curb uncontrolled deforestation.^1^

We carry our analysis across four periods of environmental policy-making in the Amazon that are representative of the struggle to use or conserve its forests. They are the state-led ‘colonization’ period of the Transamazônica/Polamazônia in the 1960s–1980s; the PPG-7/Planafloro donor-led socio-environmental management period of the 1990s; the state-led pro-development planning (Avança Brazil and Plano de Aceleração do Desenvolvimento—Programa de Aceleração do Crescimento (PAC))/soya frontier expansion era of the early 2000s; and the Avoided Deforestation/Compensated Reduction (AD/CR) phase potentially emerging today. Across these four periods, one can see the policy process in the Amazon as something of a see-saw, in which the weight of policy-making shifts from pro-development to pro-environment agendas, and back. The key to which side gains control of federal and local policy-making is the result of the players they bring into their networks: some provide numbers; others, weight.

Historically, in making environmental policy in Brazil, local and national actors battle in a complex and shifting arena, but the process has moved from being mostly an insulated technocratic and elite-dominated affair in the early 1970s to a more participatory and inclusionary process today. The diversification of the policy arena has led to the formation of dynamic policy networks that position themselves vis-à-vis different environmental problems. These fluid networks integrate domestic and global interests as well as actors across the state-society divide. Both pro-development and pro-environment networks seek to influence public opinion and claim to speak for the national or global public interest (Fearnside 2003, p. 757).

Policy networks are ‘public, semi-public and private actors participating in certain policy fields’ (Kickert *et al*. 1997). Within these networks, actors are dependent on each other to reach their goals and employ a variety of strategies to interact with the state and other organized interests (Klijn 1996; Blom-Hansen 1997). Networks are ‘lighter on their feet’ than more formal coalitions or alliances, and have been especially effective in using information and shaming in forcing changes in the behaviour of their adversaries (Keck & Sikkink 1998). Networks seek to influence both policy itself and the rules of the arenas where these policies form.

### (a) Patterns of environmental policy-making in Brazil and the Amazon: background and developmentalism

Historically, environmental policy-making in Brazil resembles a see-saw: on one side we find developmentalism that understands natural resources (including clear air and water) as the raw materials of economic growth. On the other side is socio-environmentalism that, although not necessarily denying the need for economic growth, often believes the price paid so far by Brazil's nature, indigenous peoples and the poor is too high. In practice, this see-saw is populated by different networks of domestic and international actors across the public/private divide. Membership in these networks is neither static nor exclusive and at times actors will flip from one side to the other.

In the wake of the Brazilian economic ‘miracle’ of the 1960s and 1970s, the belief that resources were infinite, and that growth was always good, was perhaps the only consensus among business, government and popular classes (Camargo 1989). Even among the opposition, criticism focused on social inequality and completely ignored its environmental consequences (Viola 1987). Brazil's patrimonial order created a policy-making apparatus in which the state was often the sole actor responsible for defining and deciding how to explore the country's natural resources. In the Amazon, the state's reach often exceeded its grasp, but the military government was able to take control of tremendous swaths of territory and land-use decisions outside these axes (Santos 1981; Bunker 1985; Schmink & Wood 1993; Roberts & Thanos 2003). This pattern of exclusionary policy-making was reinforced by the predominance of technocratic insulation through which well-placed *técnicos* within the state apparatus were able to design and implement policy, virtually unaccountable to the rest of Brazil's society (Lemos 1998). Many of the government agencies created to manage natural resources were more interested in the exploitation of these resources than concerned with conserving them or adopting sustainable development approaches.

The growth project spearheaded by the military dictatorship (1964–1985) included, first, the geopolitical concern to ‘occupy’ the Amazon region. Second, it sought to induce rapid economic growth through the building of roads such as the Transamazon and the establishment of fiscal and institutional incentives for large private- and state-owned business to acquire wide swaths of land for cattle ranching, lumbering and mining projects (Hecht 1985). At the same time, the construction of large infrastructure projects (e.g. roads, dams, etc.) acted as a magnet for large Brazilian construction firms to increase their influence on the side of development (Foweraker 1981; Pompermeyer 1984).

In 1973, the military government enacted an important piece of environmental legislation and created the Special Secretariat of the Environment (SEMA) under the supervision of the Ministry of the Interior, primarily as a way to diffuse harsh international criticism to the government's environmental performance (Viola 1987). Despite SEMA's limited range and power, the 1973 law represented a critical political opportunity for environmentalism and environmental groups to emerge as credible players in environmental policy-making. Another factor strengthening the influence of pro-environment networks was the radicalization of the Brazilian environmental movement as it became involved, together with other grass-roots movements, in the mobilization against the authoritarian regime and the design of Brazil's new democratic constitution in 1985 (Viola 1987).

As the rate of destruction of the Amazon forest skyrocketed and the level of international awareness increased, new players emerged trying to influence environmental policy-making for the region. These included the media—which periodically invited attention to environmental problems, stirred up public opinion and kept environmental issues alive in the public's mind—international and domestic NGOs, and celebrities mobilized against mega-development projects associated with high rates of forest loss.

Two of these projects were particularly critical to mobilize domestic and international support for conservation. The first project involved the development of a huge mining project, Carajás, which was financed by loans from the World Bank, private banks and prospective iron buyers around the world (Roberts & Thanos 2003; Rodrigues 2004). The project mobilized strong opposition from environmental groups and encouraged the formation of pro-environmental coalitions which included both domestic and international NGOs. The second project was Polonoroeste, also funded by the World Bank, to assist the colonization of small farmers in Rondônia state. Environmental organizations such as Friends of the Earth UK, the Environmental Defense Fund, Conservation International and the Natural Resources Defense Council brought some disturbing details to the attention of the European Commission (EC) and the US Congress about World Bank-funded ‘megaprojects’, including Carajás and Polonoroeste. The EC threatened to boycott Carajás iron if charcoal-burning pig iron smelters were built. An unusual coalition of environmentalists and fiscal conservatives in the US Congress took up the issues through the House Banking, Senate Foreign Operations and other committees who threatened the Bank's ‘replenishments’ unless it addressed detailed concerns about its projects’ environmental impacts (Keck & Sikkink 1998; Roberts & Thanos 2003; Hicks *et al*. 2008). In 1985, the Bank responded to these threats by suspending disbursements to the Polonoroeste settlement project in the Amazon.

In the western Amazon states of Acre and Rondônia, the Brazilian government responded by creating the ‘Project for the Protection of the Environment and Indian Communities’ (PMACI) which allowed them to receive funding from the World Bank and the Inter-American Development Bank for paving highway BR-364 (Woodword 1989; Allegreti *et al*. 1998). The Union of Indigenous Nations in Acre joined their former enemies, the National Council of Rubber Tappers, forming the Amazonian Alliance for the Peoples of the Forest. The assassination of rubber-tapper leader, Chico Mendes, in late 1988 brought global attention to the tensions between the Forest People's coalition on the one hand and ranchers and developers on the other. In the wake of these conflicts, the cancellation, delay or reformulation of major development projects for the region marked the first major ‘wins’ of pro-environment coalitions in redirecting developmentalism in the Amazon. They also profoundly changed the way the World Bank and other major aid agencies funded development projects in environmentally sensitive regions of the world.

### (b) Donor-led socio-environmental management: the Planafloro/Plano Piloto Grupo-7

In the 1990s, a broader consensus began to emerge on the need to work cooperatively to manage the disorder and environmental destruction in the Amazon region. Influenced by a much better organized and professionalized Brazilian environmental movement—partly as a result of the 1992 UNCED conference held in Rio de Janeiro—and by strong international mobilization, projects such as the Planafloro, Podeagro and the PPG-7 (Pilot Programme to Conserve the Brazilian Rain Forest) changed the tone and direction of the debate to diffuse some of the greatest tensions between developmentalism and conservationism in the region (Mello 2005; Redwood 2005). The new coalition for sustainable development in the Amazon included both international and domestic NGOs, supportive officials within the World Bank, western developed countries and Brazilian governments, epistemic communities of scientists, and grass-roots groups, such as rubber tappers and indigenous people.

At the 1990 G7 summit in Houston, Texas, German chancellor Helmut Kohl led an effort to develop a proposal with four goals, including conserving biodiversity, reducing rainforest contributions to climate change, demonstrating that sustainable development is possible in tropical forests, and showing that the North and South can cooperate on global environmental issues (Redwood 2005). The PPG-7 funded 181 small projects in the next 8 years, supporting the demarcation of 45 Mha of indigenous reserves and four federal extractive reserves; new participatory methods for project implementation brought in university professors and NGOs as active participants (Allegreti *et al*. 1998; Mello 2005; Redwood 2005).

These projects went far beyond technocratic management of forests: they sought to manage whole sets of people in civil society groups, the private sector and the state. They also sought to shift incentives and policies and build capacity to conserve biodiversity and provide sustainable livelihoods in the Amazon region, focusing often on agroforestry systems in suitable areas and ‘agro-ecological zoning’. Stakeholders including local, national and international development and environmental groups were invited to participate in the project's reviews (Redwood 2005, p. 103). However, ecological zoning of land use proved nearly impossible in the face of resistance from a pro-development coalition that included loggers, ranchers and farmers (Redwood 2005; Killeen 2007).

Thus the 1990s ushered in a new era in the Brazilian Amazon, which we would characterize as ‘donor-led socio-environmental management’. Some of the projects of the Planafloro/Prodeagro and PPG-7/Probio/FunBio failed to move forward or accomplish their goals because they were opposed and undermined by politicians and economic elites at the local or state levels and because they were very poorly executed (Fearnside 2003; Killeen 2007). The overall achievements of this innovative donor-led effort to manage biological and social systems at the same time were significant, but while demonstration projects and even an institutionalization of social movement organizations and research institutions occurred in the 1990s (only partly as a result of the projects), deforestation rates remained high.

### (c) Avança Brasil/Plano Plurianual PPA/PAC and the soya frontier expansion period

After outside attention on the region died down by the mid-1990s, the Brazilian government set out a 7 year, US$10–40 billion, set of projects to boost its infrastructure and exports for the Amazon. Named *Avança Brasil* (Forward Brazil), the project called for the paving of thousands of kilometres of Amazon roads, the deepening and straightening of key rivers for shipping, the construction of ports and dams, the expansion of power lines, the exploration of gas and oilfields (and construction of pipelines) and the granting of logging concessions. Much of this development was geared to provide a cheaper outlet to the sea for soya farmers in Brazil's booming Centre-West agricultural belt (Ministério do Planejamento 2001*b*). Plans to pave the old rutted dirt roads that often become impassable in the rainy season were perhaps the most contentious part of Avança Brasil. At the centre of the controversy was the BR-163, a 1970s-era dirt highway which slices into the heart of almost intact Amazon forest from Cuiabá in soya-producing Mato Grosso state to Santarém, in Pará.

If fully implemented, Avança Brasil was foreseen to have severe consequences for the Amazon forest. Scenario-based studies predicted a range of negative impacts for biodiversity, climate and natural resources conservation in the region for the following 20 years (Laurance *et al*. 2001; Soares-Filho *et al*. 2006). Although the Brazilian government and other scholars questioned the validity of these predictions (Câmara *et al*. 2005),^2^ opposition to Avança Brasil remained high until the end of the Cardoso administration (1995–2002). Many proposed projects were placed on a slower track, and the Brazilian government, mostly owing to lack of funds, quietly abandoned others. The following administration of Luis Inacio Lula da Silva replaced Avança Brasil with the Programa de Aceleração do Crescimento (PAC) with a similar developmentalist agenda for the Amazon region. And similarly to Avança Brazil, opposition soon emerged. For example, groups such as Greenpeace have launched aggressive campaigns exposing the perils of development-oriented policy including lumbering and soya agribusiness. In 2006, Greenpeace brokered both a moratorium on soya from recently cleared forests and an agreement with several European retailers not to purchase soya from Amazon grain terminals (Greenpeace 2006*a*,*b*). The environmentalist campaign led to a promise by Mato Grosso Governor and soya magnate Blairo Maggi to freeze soya frontier expansion in the state for 2 years. However, the apparent compromise solution will be the paving of BR-163, but with a new set of protected areas along the highway and no services along the road. We see this untested strategy as essentially a ‘quid pro quo’ between the two opposing policy networks, reflecting a stalemate in the process.

### (d) Trade or aid? Avoided Deforestation/Compensated Reductions proposals and the Amazon

In view of tropical deforestation's estimated role in carbon emissions—10–25% of global human-induced carbon emissions and 60% of Brazil's greenhouse gas emissions (Santilli *et al*. 2005; Ebeling 2006, 2008)—it is not surprising that a new policy network has emerged to advocate for solutions that both decrease emissions and protect the Amazon's threatened natural resources and biodiversity. Santilli *et al*. (2005, p. 270) suggest the concept of compensated reductions that would allow developing countries to participate in the Kyoto Protocol by electing to reduce their national emissions from deforestation during the 5 years of the first commitment period (taking average annual deforestation over some agreed period in the past, measured with robust satellite imagery techniques, as a baseline). Under one approach, these countries would then be authorized to issue carbon certificates, similar to the Certified Emissions Reductions (CERs) of the Clean Development Mechanism of the Kyoto Protocol that could be sold to governments or private investors. This scheme is the core of the Papua New Guinea/Coalition for Rainforest Nations (PNG/CRN) proposal, announced in late 2005.

The PNG/CRN proposal for Avoided Deforestation (AD) contains a key issue that Brazil fears, namely the provision that countries having received compensations would face mandatory future CO_2_ reductions. Brazil's historical opposition to any approaches that would help Annex 1 countries to reach Kyoto targets, at the ‘expense’ of less developed countries, has been mentioned as a factor to explain the country's reluctance to join the Coalition of Rainforest Countries. The Brazilian government defends this position based on protecting ‘the scientific integrity of the Kyoto Protocol’ (Ikeda 2007, personal communication to J.T.R.). However, there is wider agreement around the idea that opposition is more critically informed by the possibility that AD would negatively impact the price of CERs and the country's ability to attract ‘regular’ CDM projects already worth millions of dollars. Other reasons include Brazil's reluctance to forego potential future development and its fear that deforestation could become a focus of international attention to the detriment of the country's image. Finally, historical focus on conspiracy theories about the internationalization of the Amazon (Fearnside 2001*a*) may also explain resistance from some sectors of the Brazilian society to subscribe to AD as a carbon offsetting mechanism.

An array of public and private actors has emerged as stakeholders around this issue both domestically and internationally, and policy networks have formed seeking to influence the evolution of the issue in the public policy-making process. Internationally among the groups supporting AD as a carbon offsetting mechanism are large NGOs such as World Wide Fund for Nature, Conservation International, The Nature Conservancy and Environmental Defense Fund (Fearnside 2001*b*). The proposal has also been endorsed by other groups of countries such as the Pacific Island Forum, European Union, British Commonwealth, African Union and Association of Small Island States and professional organization such as the Association for Tropical Biology and Conservation (Laurance 2007). In contrast, earlier, a number of European NGOs opposed AD on the grounds that it would allow wealthy nations such as the US to ‘get away with’ not reducing their emissions if cheap carbon offsetting is an option. However, the magnitude of the problem as well as the technical improvements in the new proposal (especially in addressing ‘leakage’ of projects) has softened their opposition to AD (Laurance 2007). Domestically, groups such as the Brazilian Forum of NGOs and Social Movements for Environment and Development, focus on the opportunity AD provides for attracting critical resources for forest maintenance and community development (Fearnside 2001*a*).

Not surprisingly, domestic opposition to AD is represented by a number of familiar interests across the development/conservation axis (Fearnside 2001*a*; Santilli *et al*. 2005). The Labour Party controlled Environmental Ministry (MMA) has agreed ‘to carefully contemplate the proposal’ (Laurance 2007, p. 21). Among detractors are regional developmentalist interests supporting the economic growth plans and nationalist sectors within the Brazilian government concerned both with sovereignty issues and potential loss of future development options (Laurance 2007) as well as possible negative effects of forest-based schemes in threatening Brazil's ability to attract CDMs and the technology transferred through these projects (Ebeling 2006, 2008; Hall 2008). Within the Amazon, another set of actors is likely to arise in opposition to AD/CR schemes which have been little explored and discussed so far (Costa 2007). Compensating land users to not produce on a piece of land will create major (negative) multiplier effects in the local economy, Costa argues, by reducing the opportunities for employment created by processing these local products (Costa 2007).

In this scenario, international support and political opportunity have a critical role to play in the way these networks go about pushing for the conflicting policy agendas. The global character of the climate change issue coupled with the formalization of its global governance, may provide the environmentalist network with an opportunity to build international support and pressure opposing interests within Brazil, similar to what happened in the cases of the Polonoroeste and Carajás programmes 20 years before. However, this approach can backfire if it stirs entrenched fears in Brazil's society against the ‘internationalization’ of the Amazon.^3^ In order to be successful, any AD proposal will have to be ‘backed up’ by concrete benefits that compensate for what is perceived as foregone opportunities for development and strongly shift economic incentives and provide local social development (Killeen 2007; Hall 2008). What this means for distributing compensation, especially at the local level, remains entirely unspecified; we discuss this point further in the concluding section.

### (e) Incentives, compensation and policy networks: analysis/conclusion

Through the four stages of Amazon history, the overall trend has been one of continuing deforestation, but the situation and players have changed in important ways. The networks forming around the issue of climate/forests are constantly repositioning themselves in terms of political opportunity, international pressure and organizational resources.

In this context, the World Bank has played an important role as it has moved from one side (funding settlements in Rondônia, along the Transamazon Highway and the Carajás mining project in the 1980s) to the other (sustainable agroforestry and participatory projects in the 1990s). The slow political opening of Brazil to relative democracy in the 1980s also played a decisive part in allowing an opportunity for environmentalists, rubber tappers and indigenous peoples to organize. In spite of high expectations from citizens' groups, the rise of the Workers' Party represents only an ambiguous window of opportunity for environmentalism and a new model in the Amazon. While some of the projects of the Avança Brasil/PAC proposal have been quietly moved off the front burner, the paving of the BR-163, the construction of the Madeira dams and other elements of opening the region to global trade (especially exports such as soya) remain in place under the new government.

The AD proposal represents a clear split in bureaucracies in the Brazilian state and potentially a major shift that might bring billions of dollars to the region in return for serious efforts to slow the devastation. The AD/CR proposal is potentially important globally because it represents the first time that ‘losers’ in the climate regime will be directly compensated for foregone economic gains from their helping the world address this problem. However, many uncertainties characterize the design of an effective institutional arrangement that meets carbon offsetting and conservation goals, while having a realistic chance of blocking developmentalist networks’ opposition. Part of the problem is what sectors and actors are slated to win or lose if AD/CR is implemented. Some observers fear that national, state or local bureaucracies or even corrupt politicians would siphon off a substantial proportion of the funds, for example. Others worry that such schemes will fail to address the poverty and inequalities which underlie small farmers' and colonists' reasons to continue felling forested areas, and the desperation of Brazil's poor that provides cheap and exploitable labour on the frontier.

In conclusion, we would argue that international influences and fiscal resources have been substantial in determining the outcome of the four different phases, but in the end they are nearly always outweighed by Brazilian actors in the developmentalist policy network. This historical analysis suggests that developmentalist blocking coalitions will be unlikely to be diffused until economic incentives are strongly reversed and they perceive pro-environment schemes as a potential source of resources. Transparency in accounting and distribution of any funding to come for AD/CR is crucial, and funding must get down to the local people who will be asked to forego the income they would have made from selling logs, soya, beef or other products produced on rainforest land, or from wages foregone from the halting of such production. As Costa points out, different types of land users may be giving up different probable incomes by agreeing not to produce on a piece of land (2007). Furthermore, stopping or sharply reducing agricultural production in a region is likely to have a series of consequences that must be considered for such a plan to succeed. The earliest distributions of AD/CR funds will be critical in either supporting a positive model for this programme or spreading cynicism in the Amazon. And while international actors play an important role in Amazon policy-making, their influence is uncertain for the AD/CR case, since, as always, Brazil will have the last say and will restructure the bargain to its advantage.

## Endnotes

1 Within Brazil, groups that support both environmentalism and indigenous and popular movements causes are known as socio-environmentalists.

2 Just 5 days after the *Science* article was published, Brazil's Secretary of Planning and Strategic Investments, José Paulo Silveira, held a press conference to state that *Avança Brasil* did not include 1 km of new roads and the waterways were chosen to be developed for their lesser impact (Ministerio de Planejamento 2001*a*). Silveira said, ‘The predictions of deforestation are exaggerated and do not reflect a more careful analysis of future scenarios, rather, they are based on data from 20 years ago before Brazil had environmental laws’.

3 For example, in October 2006, Marina Silva and Celso Amorim, Ministers of Environment and International Relations, respectively, in an open editorial in the Folha de São Paulo, vehemently denounced any plans to ‘internationalize the Amazon’ coming from western countries (in this case, the UK). Folha de Sao Paulo, ‘A Amazônia não está a venda’, 17 October 2006.
